# Endoscopic direct-vision therapy for a foreign body in appendiceal lumen in one patient with acute right-sided abdominal pain

**DOI:** 10.1055/a-2816-3548

**Published:** 2026-04-27

**Authors:** Lifeng Liu, Zhonghua Du, Zhu Liu, Luan Kou, Fengyu Gao

**Affiliations:** 1 Department of Gastroenterology 584299 Shandong Provincial Maternal and Child Health Care Hospital affiliated to Qingdao UniversityJinanShandongChina

Endoscopic direct-vision appendicitis therapy (EDAT) represents a novel endoscopic approach for managing acute uncomplicated appendicitis 1 2 . It technically stands out for direct visualization and targeted treatment without radioactivity 3 . Herein, we present a case of EDAT for a foreign body in the appendiceal lumen in a woman with acute right-sided abdominal pain ( Video 1 ).

EDAT for the management of a foreign body in the appendiceal lumen with acute pain in a 37-year-old woman. EDAT, Endoscopic direct-vision appendicitis therapy.Video 1

**Fig. 1:**
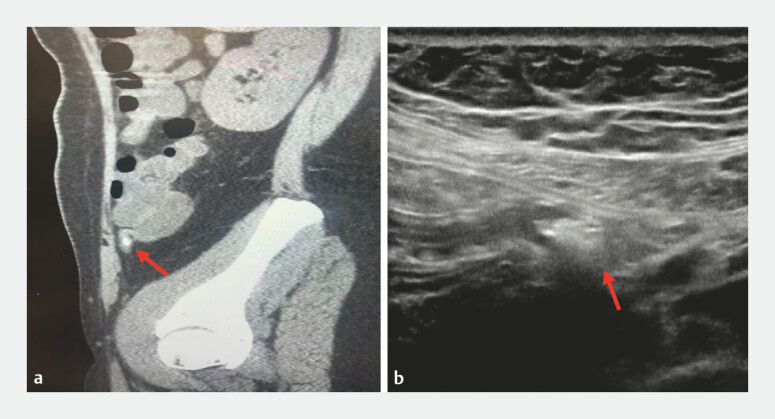
Abdominal CT **a** and appendiceal ultrasound **b** showed a foreign body in the appendiceal lumen (where the red arrow is pointing, and the diameter is 7 × 4 mm). CT, computed tomography.

**Fig. 2:**
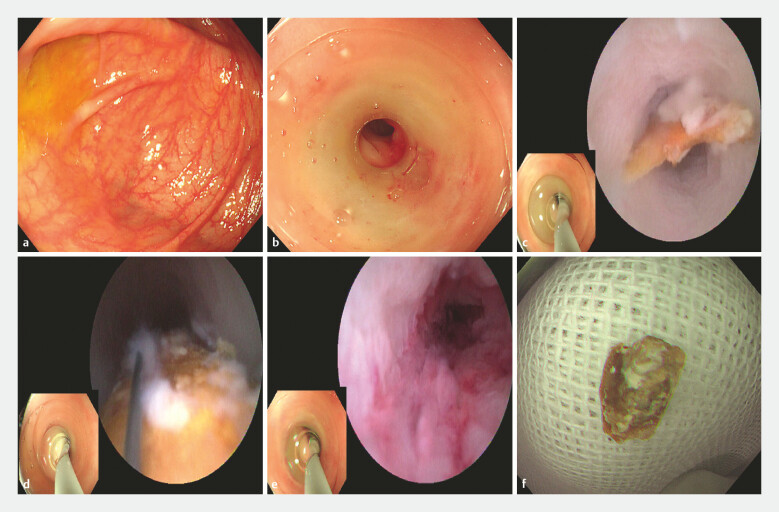
EDAT process. **a** A colonoscope was advanced to the ileocecal region, and the appendiceal orifice was identified. **b** A colonoscope with a transparent cap at the front was inserted again, and the transparent cap assisted in fixing the appendiceal orifice. **c** An eye-max was accessed through the biopsy channel and upward into the appendiceal lumen. A foreign body (diameter 7 mm) was visualized in the proximal lumen. **d** A reticular basket was inserted into the appendiceal lumen and the foreign body was extracted. **e** An eye-max was inserted again to confirm that the foreign body was cleaned completely. **f** The foreign body. EDAT, Endoscopic direct-vision appendicitis therapy.

A 37-year-old woman was admitted to our hospital, who presented with right-sided abdominal pain for 11 hours. Both abdominal computed tomography and appendiceal ultrasound showed a foreign body in the appendiceal lumen (the diameter is 7 × 4 mm) and appendicitis was considered ( Fig. 1 **a** and **b** ). We performed EDAT ( Fig. 2 **a–f** ). A foreign body blocked the proximal lumen, and it was successfully extracted through a retrieval basket. The appendiceal obstruction was relieved and the pain disappeared immediately. Intravenous antibiotics were administered for 24 hours after EDAT, and a liquid diet was initiated 6 hours postoperatively. The patient was discharged 48 hours postoperatively.

Endoscopy_UCTN_Code_TTT_1AQ_2AH
